# Authors’ Response to Peer Reviews of “A Framework for a Statistical Characterization of Epidemic Cycles: COVID-19 Case Study”

**DOI:** 10.2196/27537

**Published:** 2021-03-18

**Authors:** Eduardo Atem De Carvalho, Rogerio Atem De Carvalho

**Affiliations:** 1 Center for Science and Technology Universidade Estadual do Norte Fluminense Campos Brazil; 2 Innovation Hub Instituto Federal Fluminense Campos Brazil

**Keywords:** COVID-19, pandemics, infection control, models, experimental, longitudinal studies, statistical modeling, epidemiology

*Authors' response to peer reviews for “A Framework for a Statistical Characterization of Epidemic Cycles: COVID-19 Case Study”.*

## Response to Round 1 Reviews:

Dear Editor, we want to thank you for the opportunity offered by this prestigious journal.

### Reviewer H:

#### General Comments

We appreciate the comments on our paper [1] from Reviewer H [2], which we address point by point. Regarding the size of the paper, we selected the most significant cases and the results obtained for Brazil, reducing the length in general. Regarding the wording, the paper underwent an initial edit after the first review round and was completely reviewed after its final acceptance. In order to highlight its scientific contribution, a bibliographical review on similar and recent articles was carried out, pointing out the need to provide a simple and at the same time effective model for analyzing epidemic curves. Additionally, we reorganized the paper to make its methodological section clearer and added statistical tests that provide support to our claims.

#### Specific Comments

##### Major Comments

1. As explained before, we focused on the most significant cases and removed the items cited by the reviewer as excessive.
2. We cited in the review an article that shows how often the Gaussian models failed in the predictions [3]. That section was removed, contributing to the reduction in the length of the paper.
3. As answered in the previous item, other authors already show that classic models have failed in their predictions. What we are looking for is a simple model that can be used by health authorities, and at the same time be computationally efficient. In the text, using the appropriate reference [4], we show that the triangular distribution can replace the Gaussian distribution and its derivatives. In addition, we carried out the appropriate Kolmogorov-Smirnov tests, which prove this hypothesis mathematically for the cases studied. Therefore, we believe that we are contributing to the expansion of knowledge by including a frequency distribution that is still relatively unused in the field.
4. Citations have been properly formatted and organized.

### Reviewer X:

#### General Comments

The authors are grateful for the comments of Reviewer X [5], which were all addressed point by point below. Regarding the structure of the paper, it now follows a format that includes a structured abstract, introduction, methods, results, and conclusions, highlighting its scientific aspects. Additionally, a brief bibliographic review was included that contributes to the justification of the paper’s scientific contribution; statistical tests to assess the quality of the proposed model were also provided. In relation to the correct use of the terms of the field, the authors conducted a review and corrected them accordingly.

#### Specific Comments

1. The aim of the study is now clearly stated in the first two paragraphs of the Introduction section. A hypothesis was successfully tested using the Kolmogorov-Smirnov method in the Results section.
2. The subtopic “Nondimensional Characteristics of Epidemic Cycles” introduces the triangular distribution, its assumptions, and its limitations in the context of the COVID-19 pandemic. It is complemented by the subtopic “Predictability by Similarity.”
3. In the Methods section, the source and date of data collection are explicitly stated (Worldometer’s COVID-19 portal, as of July 9, 2020). A copy of this data has now been provided in a separate Excel spreadsheet.
4. The terms were checked and fixed; to the best of our knowledge, they now match those used by experts in the field.

## Response to Round 2 Reviews:

Regarding the journal instructions: “Using the structure used in this paper, please consolidate manuscript 23997 and 23998 into one cohesive narrative, taking into account peer-review feedback provided by the reviewers on those submissions. This way, we can present one paper with your aggregated findings in JMIRx.” We followed these recommendations and integrated the three articles originally called “Identification of Patterns in Epidemic Cycles and Methods for Estimating Their Duration: COVID-19 Case Study,” “COVID-19: Time-Dependent Effective Reproduction Number and Sub-notification Effect Estimation Modeling,” and “COVID-19: Estimation of the Actual Onset of Local Epidemic Cycles, Determination of Total Number of Infective, and Duration of the Incubation Period” into a single narrative. The resulting paper represents not only the combination of the content of the three others but an integrated narrative that describes the statistical framework developed by us to analyze the epidemic cycles. Thus, given that the resulting content reflects this integrated work, we find it more coherent to change the title of the article to “A Framework for a Statistical Characterization of Epidemic Cycles: COVID-19 Case Study.” In order to make the resulting text more fluid, we concentrated on the data analyzed in three countries (Germany, Italy, and Sweden), leaving the case studies related to Brazil to Multimedia Appendix 3. Other cases originally studied during the development of the statistical framework can still be found in the preprints duly referenced in the text. In addition, all the data obtained from the referred public databases, as well as all the calculations carried out both in the main paper and in Multimedia Appendix 3, are organized in three different worksheets (Multimedia Appendices 2-4), in order to facilitate the verification and reproduction of the results by readers.
